# Supplementary material for: Prodrug-conjugated tumor-seeking commensals for targeted cancer therapy
Source: Nat Commun. 2024 May 21;15:4343. doi: 10.1038/s41467-024-48661-y (PMC11109227; doi:10.1038/s41467-024-48661-y)
Supplement: Supplementary file 1 — Supplementary Information [file 41467_2024_48661_MOESM1_ESM.pdf]

Supplementary Information

Prodrug-Conjugated Tumor-Seeking Commensals for Targeted Cancer Therapy

Haosheng Shen[1,2,3], Changyu Zhang[4,5], Shengjie Li[1,6], Yuanmei Liang[1,2,3,7], Li Ting Lee[1,2,3], Nikhil Aggarwal[1,2,3], Kwok Soon Wun[1,2,3,7], Jing Liu[8], Saravanan Prabhu Nadarajan[1,2,7], Cheng Weng[5], Hua Ling[1,2,7,#], Joshua K. Tay[1,2,9], De Yun Wang[8], Shao Q. Yao[5], In Young Hwang[1,2,^,*], Yung Seng Lee[1,2,10], and Matthew Wook Chang[1,2,3,7,*]

[1] NUS Synthetic Biology for Clinical and Technological Innovation (SynCTI), National University of Singapore, Singapore
[2] Synthetic Biology Translational Research Programme, Yong Loo Lin School of Medicine, National University of Singapore, Singapore
[3] National Centre for Engineering Biology (NCEB), Singapore
[4] Ningbo Institute of Dalian University of Technology, Ningbo, China
[5] Department of Chemistry, National University of Singapore, Singapore
[6] Institute of Translational Medicine, Jiangxi Medical College, Nanchang University, China
[7] Department of Biochemistry, Yong Loo Lin School of Medicine, National University of Singapore, Singapore
[8] Department of Otolaryngology, Infectious Diseases Translational Research Program, Yong Loo Lin School of Medicine, National University of Singapore, Singapore
[9] Department of Otolaryngology-Head and Neck Surgery, National University of Singapore, Singapore
[10] Department of Paediatrics, Yong Loo Lin School of Medicine, National University of Singapore, Singapore

[#]Present address: Wilmar International Limited, Singapore
[^]Present address: Food, Chemical and Biotechnology, Singapore Institute of Technology, Singapore

*Correspondence:
In Young Hwang, iyhwang@nus.edu.sg
Matthew Wook Chang, bchcmw@nus.edu.sg

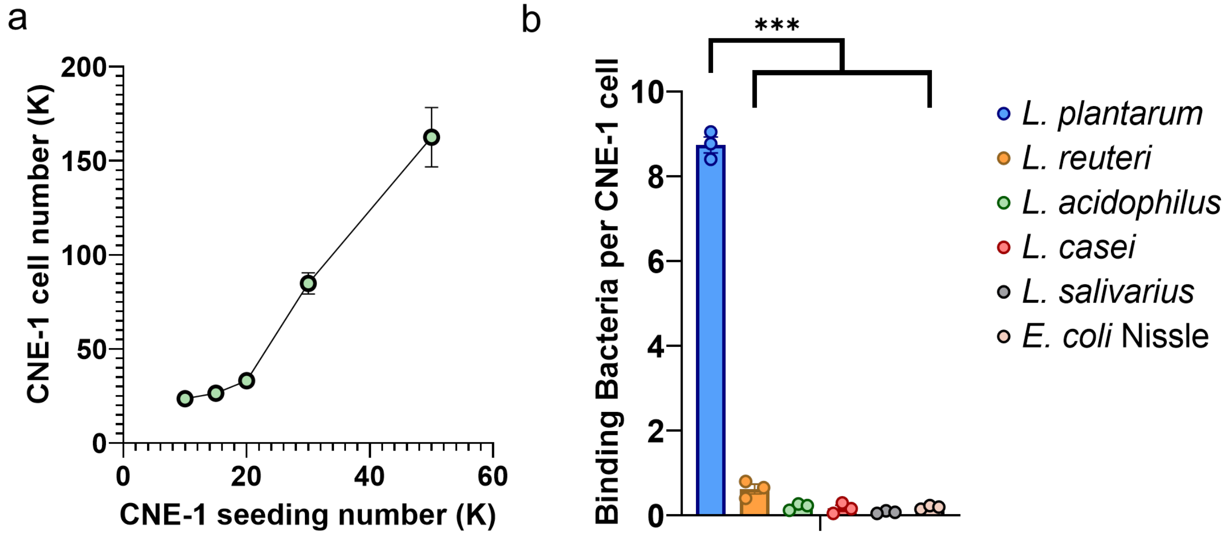

Supplementary Figure 1. Binding affinity of *L. plantarum* WCF1 toward NPC cells. a) CNE-1 cell number 24 hours post seeding. n=3 independent CNE-1 cultures. b) Number of bound bacteria per CNE-1 cell in coculture with the CNE-1 cell monolayer. n=3 independent CNE-1 cultures. All Data are presented as mean values +/- SEM. P values *L. plantarum vs L. reuteri* = 3.22×10^{-6}, *L. plantarum vs L. acidophilus* =1.55×10^{-6}, *L. plantarum vs L. acidophilus* = 1.78×10^{-6}, *L. plantarum vs L. salivarius* = 1.33×10^{-6}, *L. plantarum vs E. coli Nissle* = 1.43×10^{-6}.

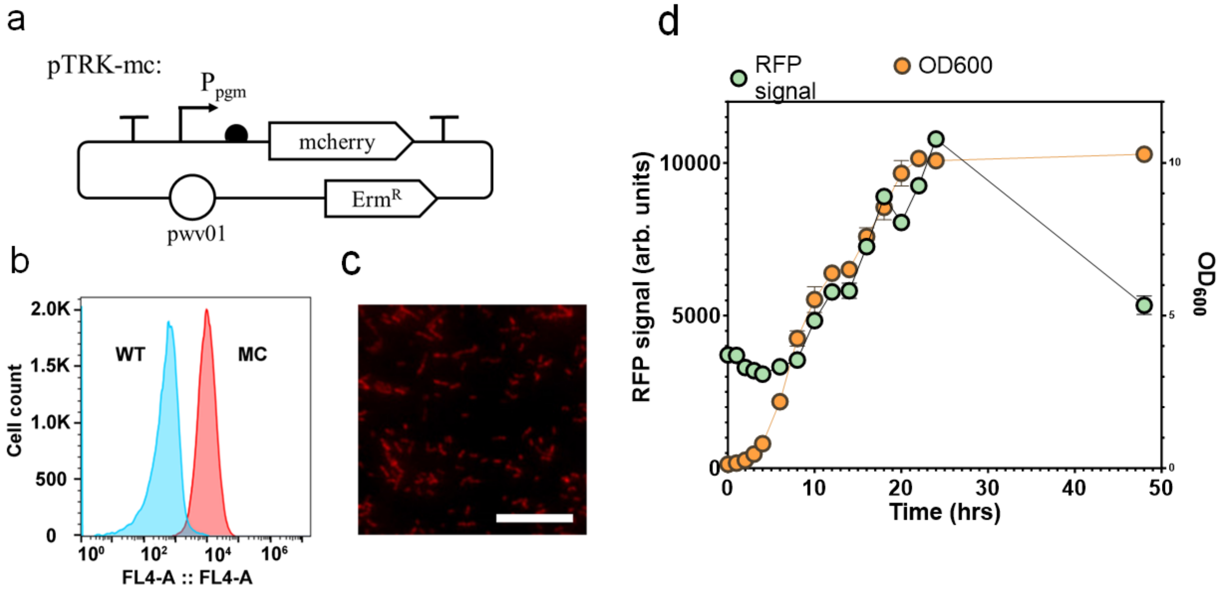

Supplementary Figure 2. Expression of mCherry in Lp. a) Expression plasmid for mCherry. b) & c) Flow cytometry analysis (n=10,000 events) and fluorescence microscopy image showing mCherry expression in Lp. Scale bar -- 25 µm. d) Growth-dependent expression of mCherry in Lp. n=3 independent Lp cultures. All Data are presented as mean values +/- SEM.

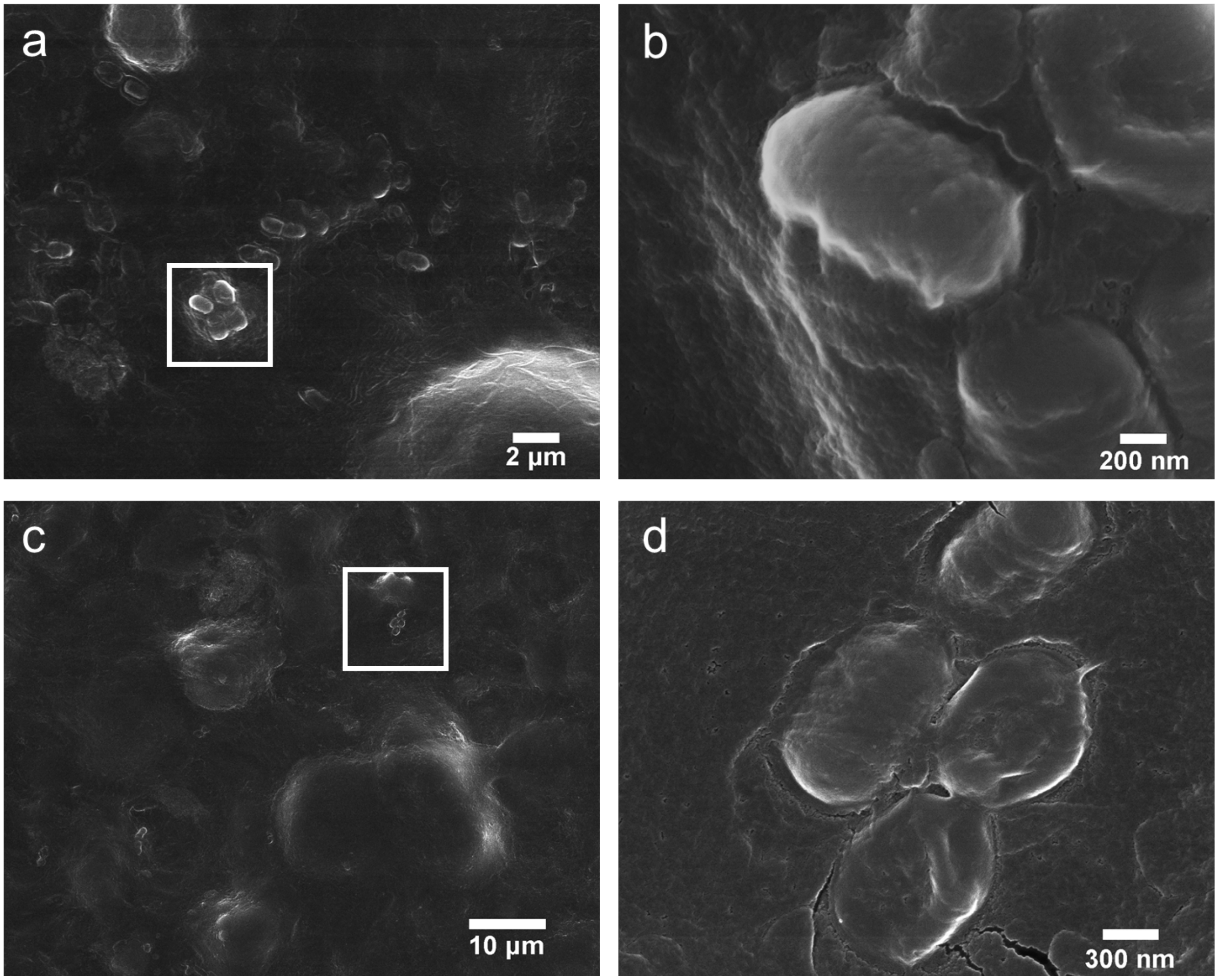

Supplementary Figure 3. SEM images of Lp cocultured with the CNE-1 monolayer. a) & c) Lp identified on the surface of CNE-1 cytoplasm at low magnification. b) & d) Presentation of Lp from a) & c) at higher magnification.

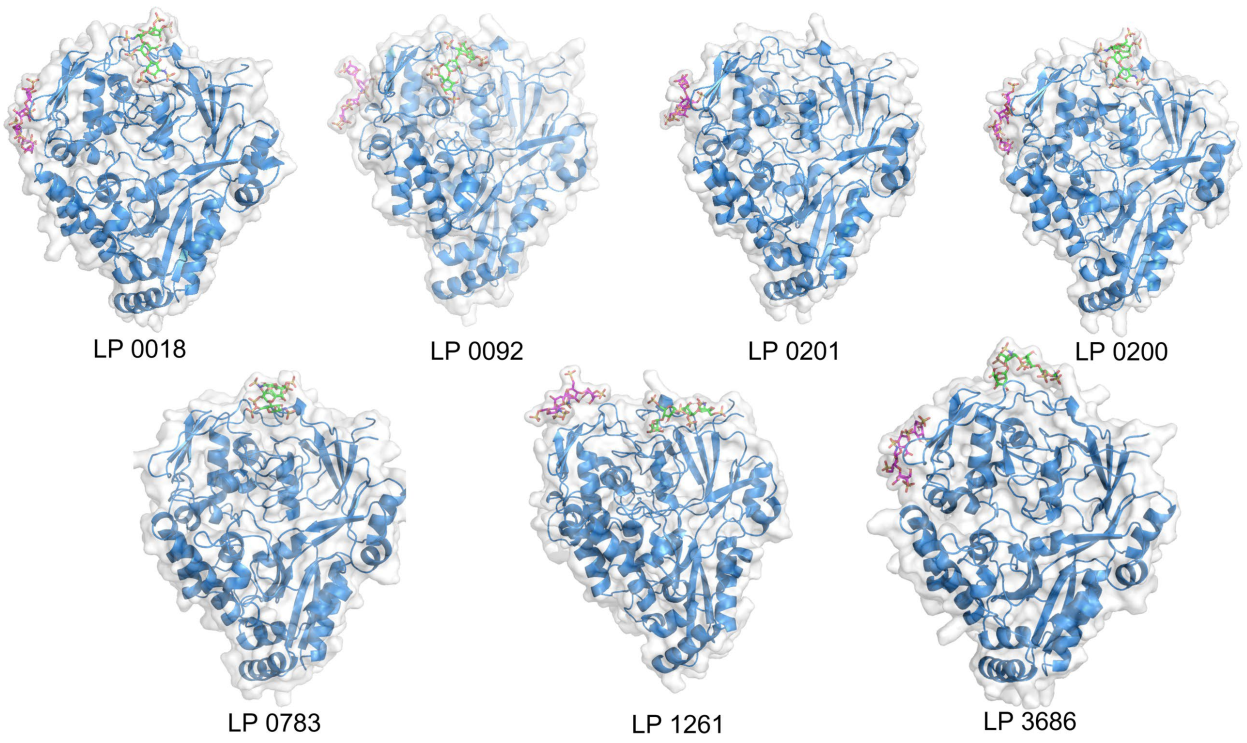

Supplementary Figure 4: Docked complexes of different OPPA proteins exerting two different binding modes. Tetramer heparin complexed with OppA proteins at binding modes 1 and 2 are represented as green and magenta sticks, respectively. Protein structures are represented in blue cartoon with white surface representation.

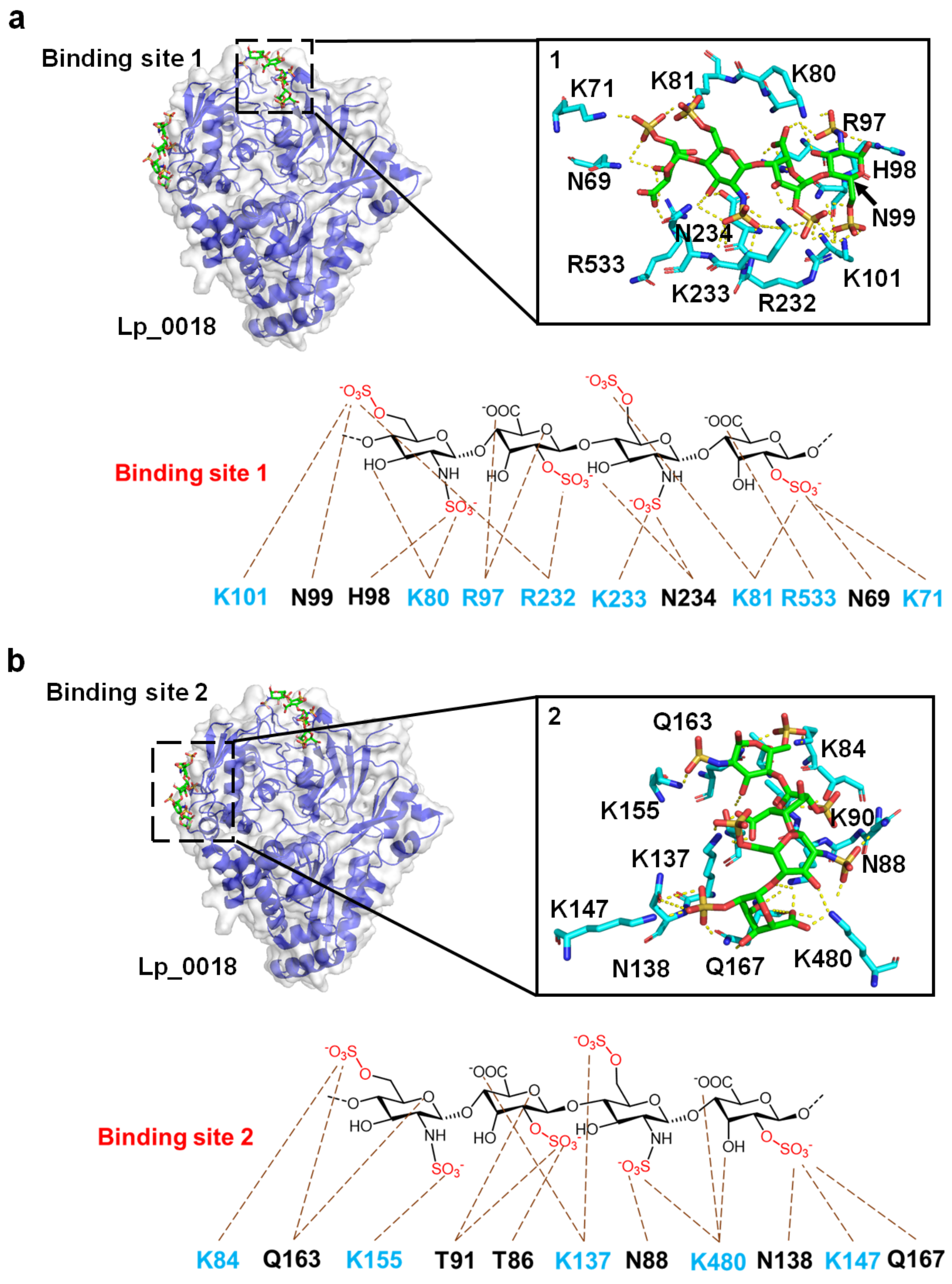

Supplementary Figure 5: Hydrogen bond interaction map for the two Lp_0018-heparin complexes. a) & b) Presentation of Lp_0018-hearin interaction at two independent binding sites. The three-dimensional structure of Lp_0018 is demonstrated in the blue ribbon model, and the heparin tetramer is shown in the green stick model. Close interacting amino acids are shown as blue sticks. Hydrogen bonds are shown as dotted lines, with the maximum distance at 3.6 Å.

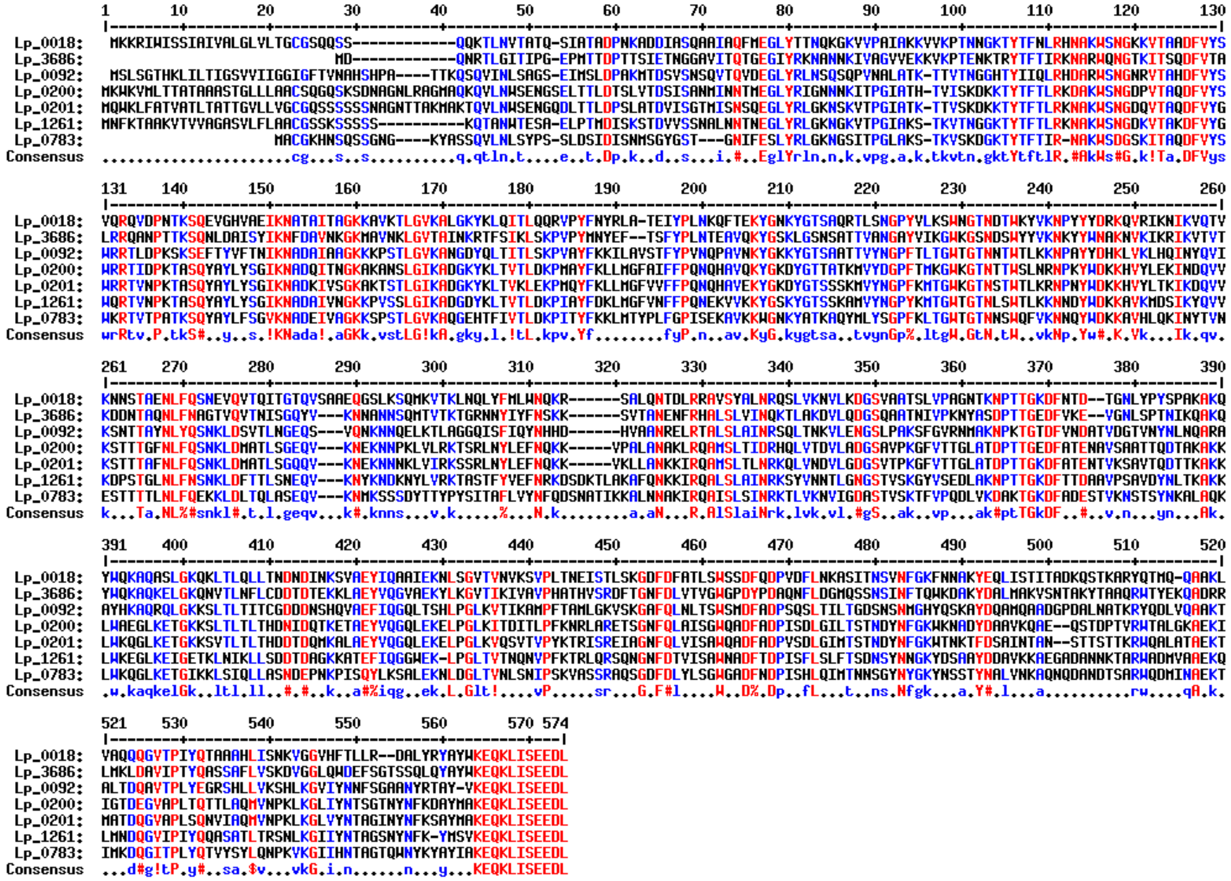

Supplementary Figure 6: Multiple sequence alignment of the seven-heparin binding protein. Highly conserved residues are represented as red upper-case letters; weakly conserved residues are represented as blue lower-case letters; and other residues are represented in black.

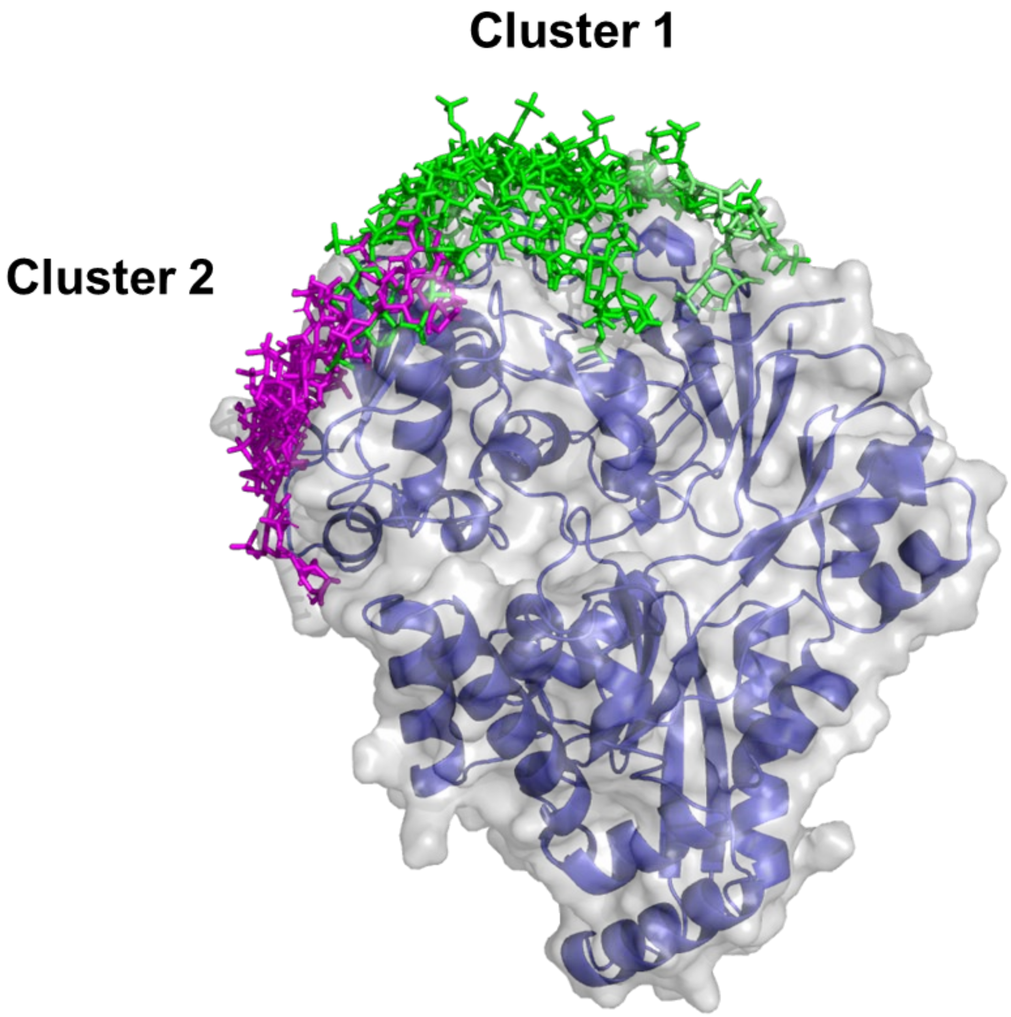

Supplementary Figure 7: Superimposed structures of Lp_0018-heparin complexes demonstrating two possible binding sites.

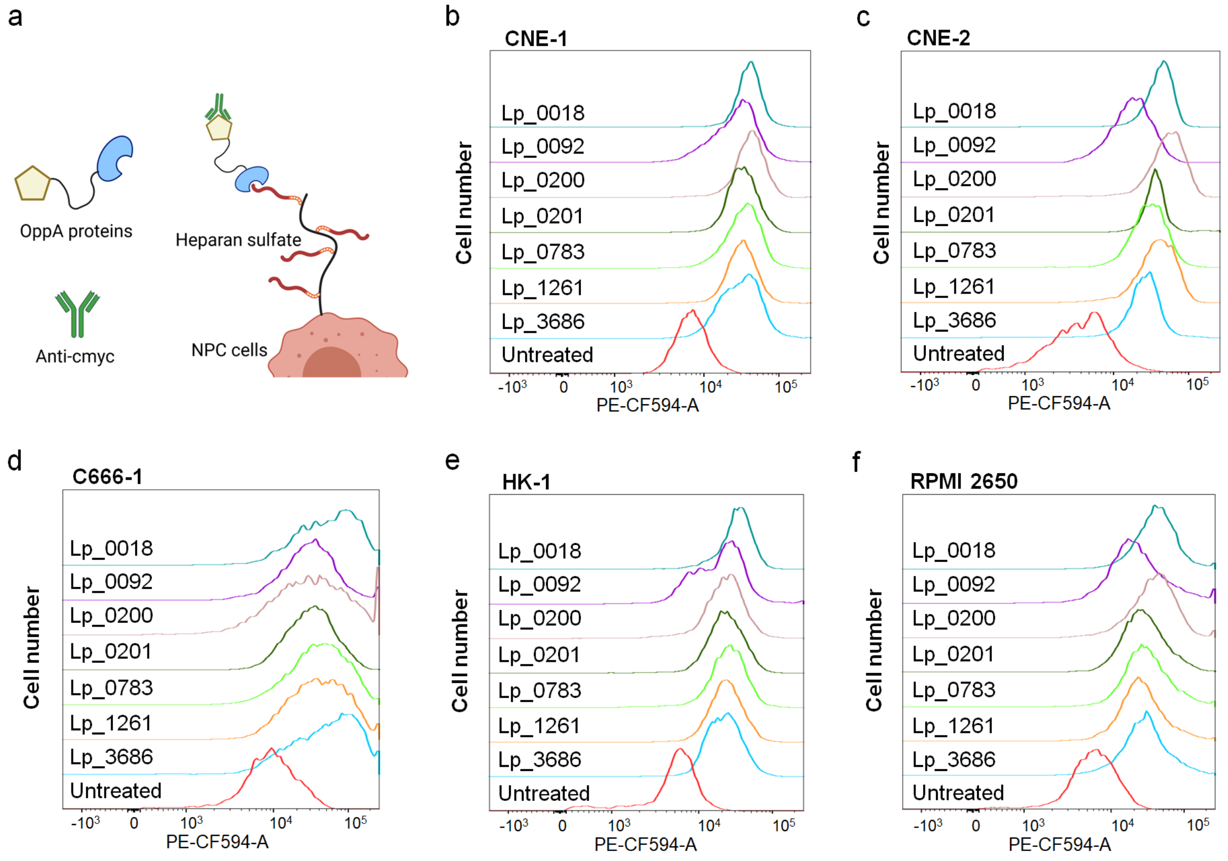

Supplementary Figure 8. Flow cytometry analysis of OppA protein binding to NPC cells. a) Schematic diagram showing the detection of OppA proteins bound to NPC cells. b) – f) OppA proteins binding to CNE-1, CNE-2, C666-1, HK-1 and RPMI 2650 cells. Whole cell population was analyzed and the geometric means of the treated cells were used to compare the binding efficacy of OppA proteins to various NPC lines. n=10,000 events.

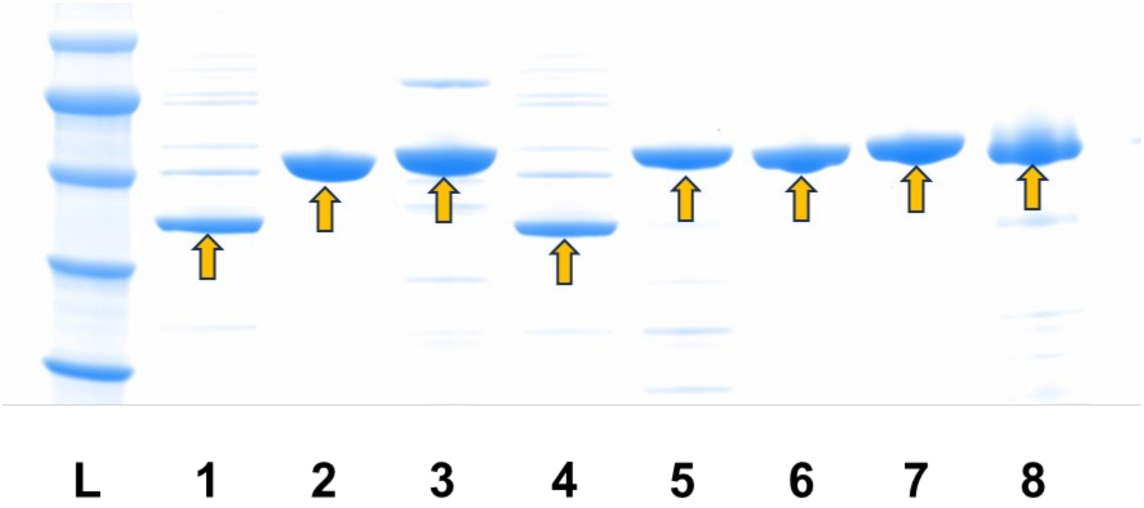

Supplementary Figure 9. OppA proteins purified through a heparin affinity column. From lane 1 to 8: Lp_0018 SBD, Lp_0018, Lp_0092, Lp_0200 SBD, Lp_0201, Lp_0783, Lp_1261, Lp_3686.

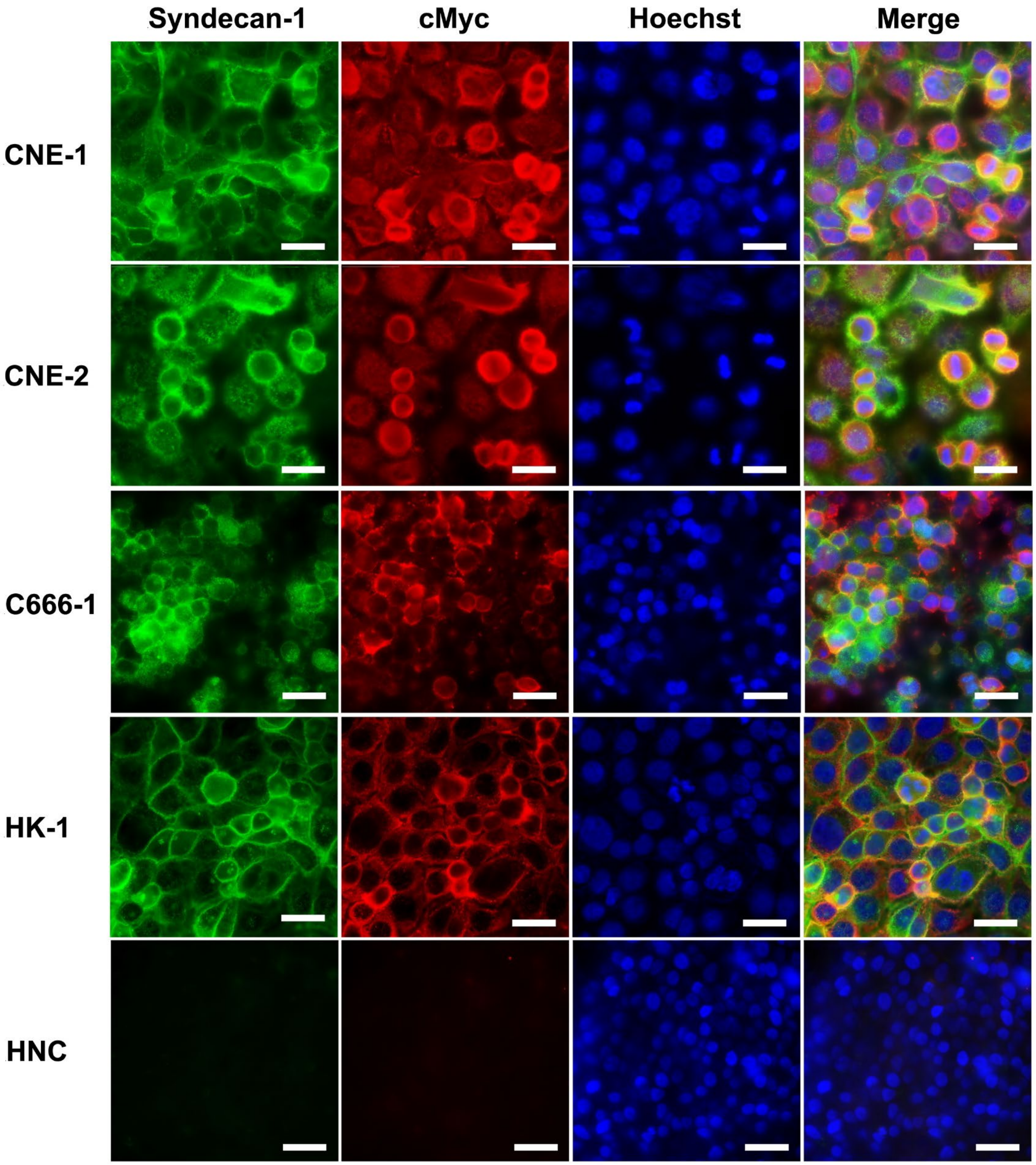

Supplementary Figure 10. IF staining showing the expression of syndecan-1 and the binding of Lp_0018 to NPCs and HNC. Green – syndecan-1, Red – Lp_0018 stained by anti-cMyc antibody. Blue -- mammalian nucleus stained by Hoechst., Scale bar -- 25 µm.

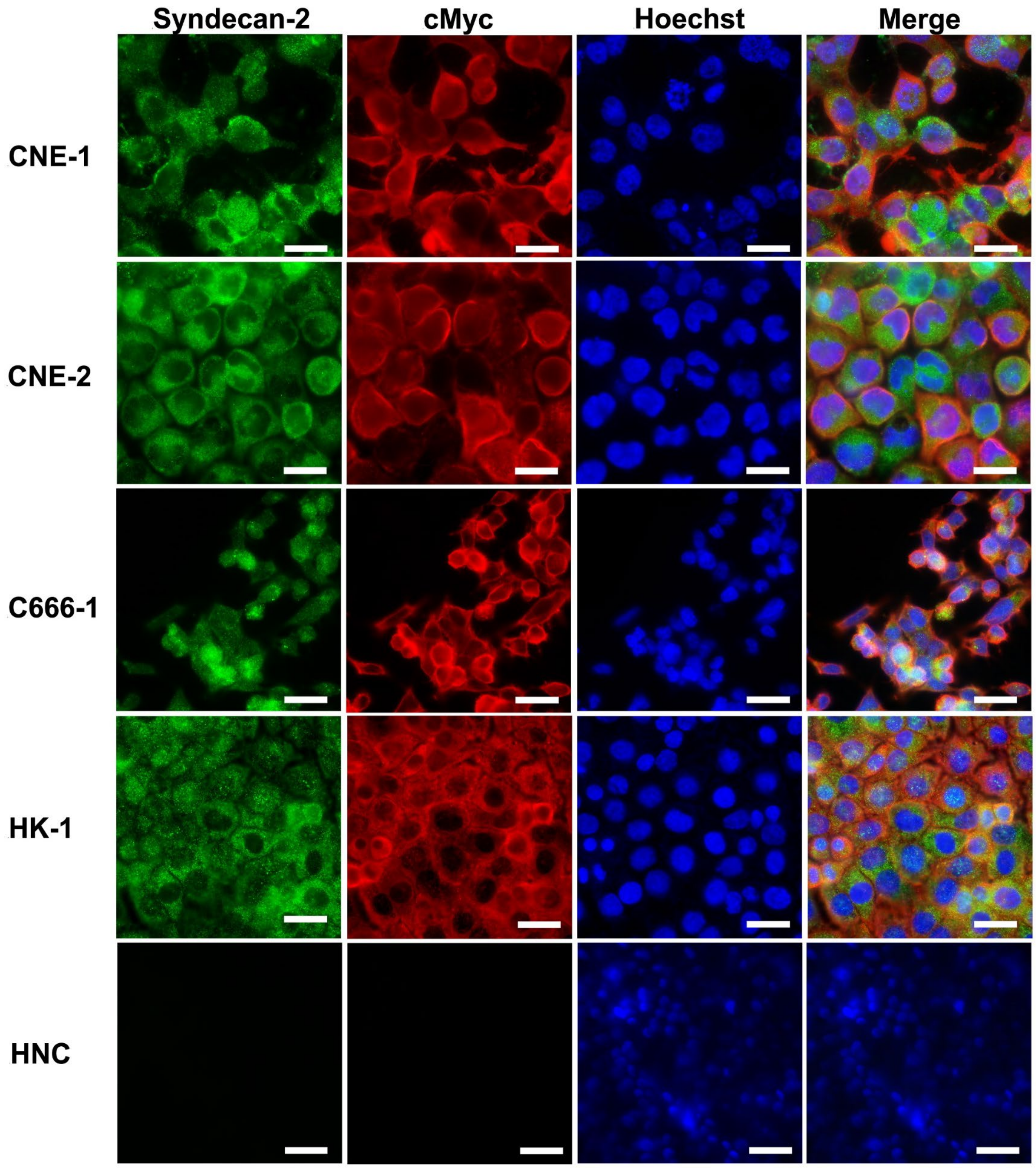

Supplementary Figure 11. IF staining showing the expression of syndecan-2 and the binding of Lp_0018 to NPCs and HNC. Green – syndecan-1, Red – Lp_0018 stained by anti-cMyc antibody. Blue -- mammalian nucleus stained by Hoechst., Scale bar -- 25 μm.

a

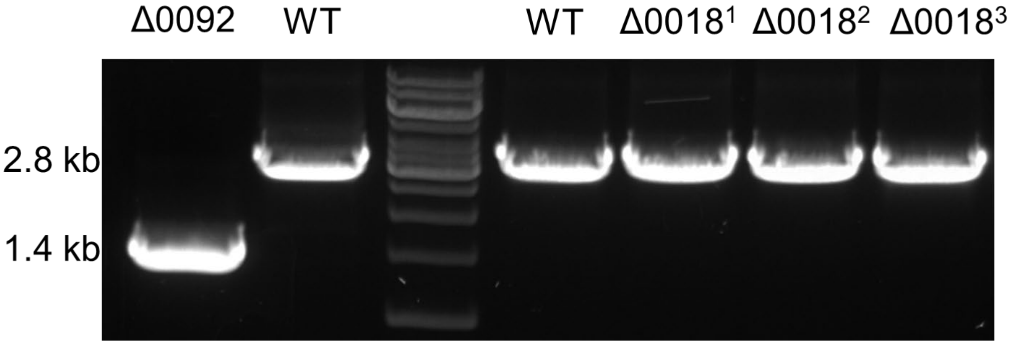

b

Strain name	Guide RNA sequence
Δ0092	CAAGUCACCCAAGUUGACGA
Δ0018[1]	GGCCUGACUAGCGAUAUCGU
Δ0018[2]	AGUAUUGCUAUUGUAGCCCU
Δ0018[3]	GCCAGUUAACACUAAUCCUA

c

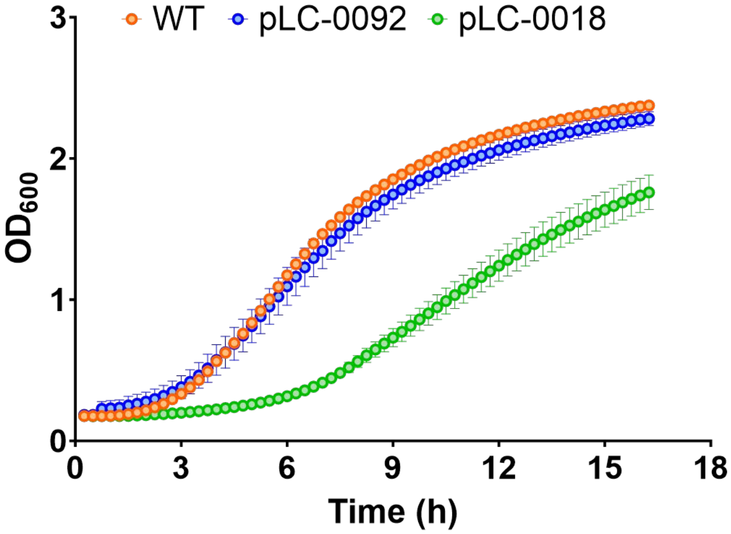

Supplementary Figure 12. Deletion of Lp_0018 and Lp_0092. a) Successful deletion of Lp_0092 and unsuccessful delection of Lp_0018. b) Guide RNA sequences used for the deletion of Lp_0018 and Lp_0092. c) Growth curve of Lp strains bearing the plasmid pLC-0092 and pLC-0018 for Lp_0092 and Lp_0018 deletion. n=3 independent Lp cultures. Data are presented as mean values +/- SEM.

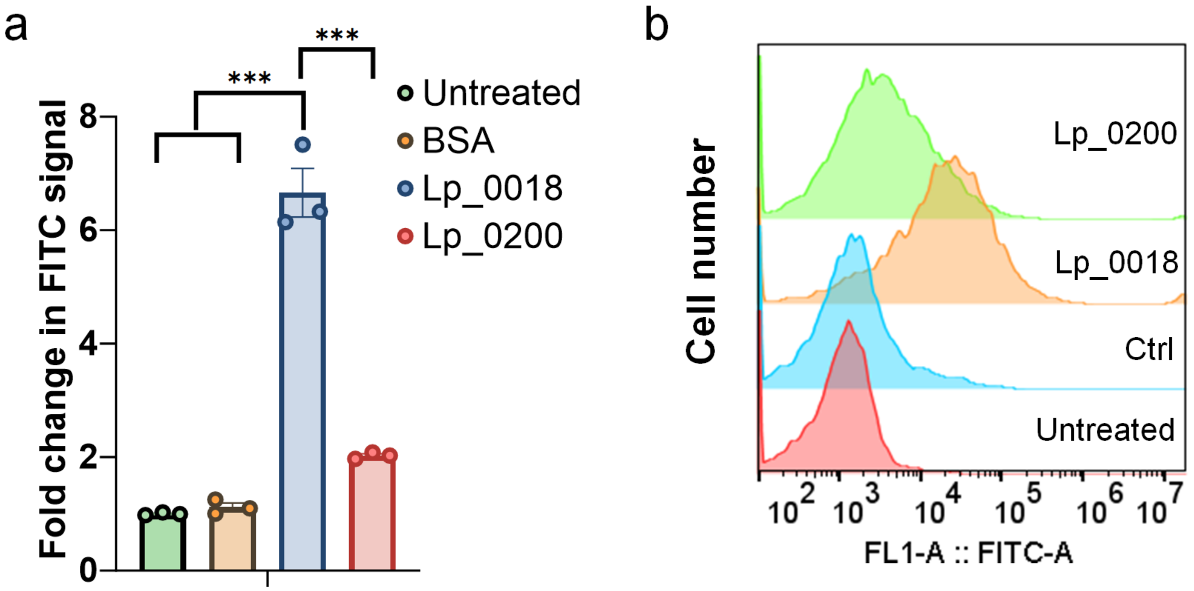

Supplementary Figure 13. Enhancement of NPC binding capacity by Lp_0018 and Lp_0200 SBD. a) Enhanced binding capacity to CNE-1 cells in Lp via incubation of Lp_0018 and Lp_0200. n=3 independent CNE-1 cultures. Data are presented as mean values +/- SEM. (P values Lp_0018 vs Untreated = 0.0002, Lp_0018 vs BSA = 0.0002, Lp_0018 vs Lp_0200 = 0.0004). b) Flow cytometry analysis showing Lp_0018 and Lp_0200 SBD binding to Lp cells. n=10,000 events.

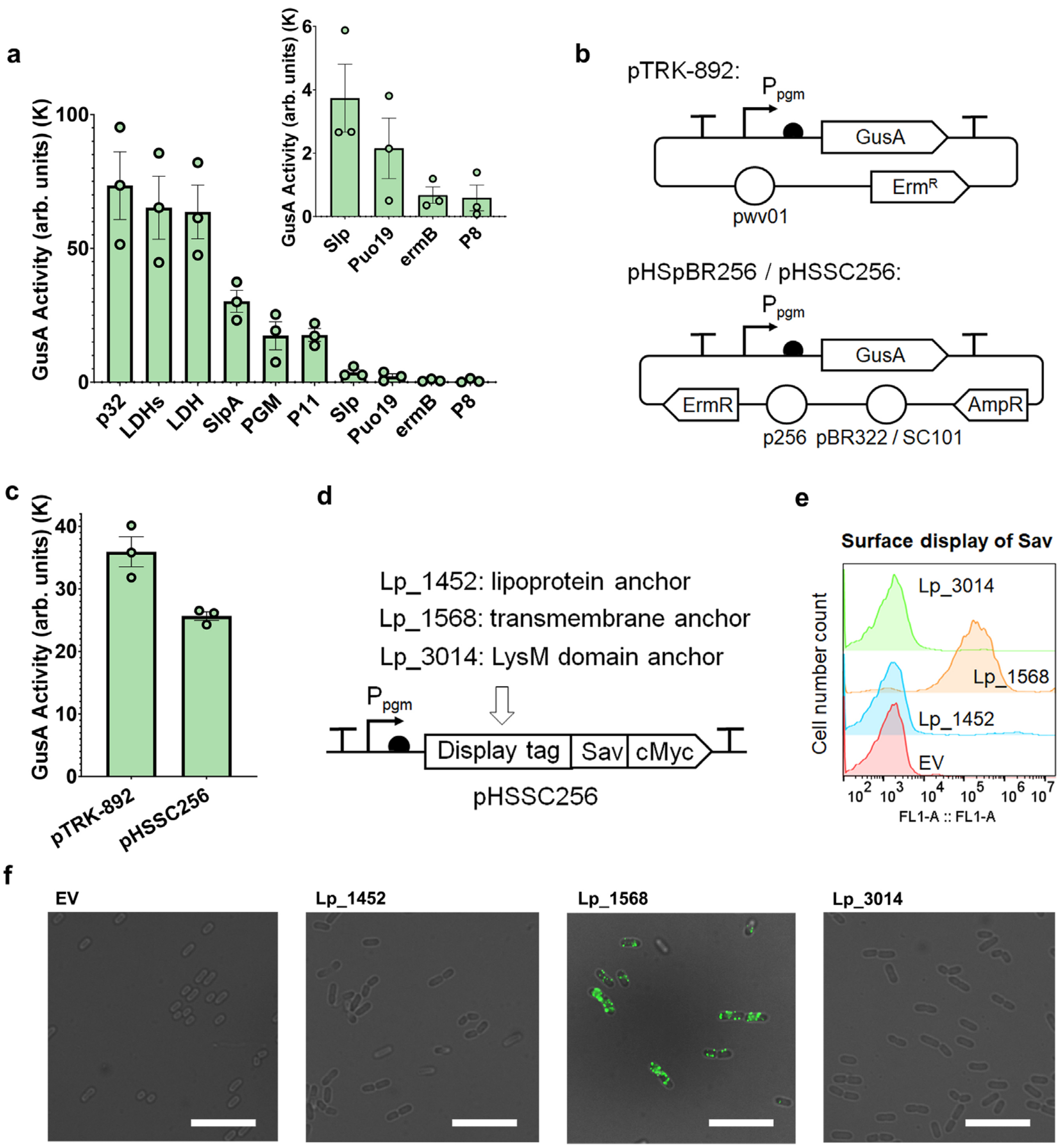

Supplementary Figure 14. Surface display of Sav in Lp. a) Promoter library in Lp characterized by gusA assay. n=3 independent Lp cultures. b). Replicon 256-based plasmids for genetic manipulation in Lp. c) Comparison of expression strength in pTRK-892 and pHSSC256 plasmids. n=3 independent Lp cultures. d) Expression cassette for surface display of Sav. e) & f) Flow cytometry analysis (n=10,000 events) and IF staining showing successful display of Sav through fusion to the transmembrane protein Lp_1568. Scale bar, 25 μm. All data are presented as mean values +/- SEM.

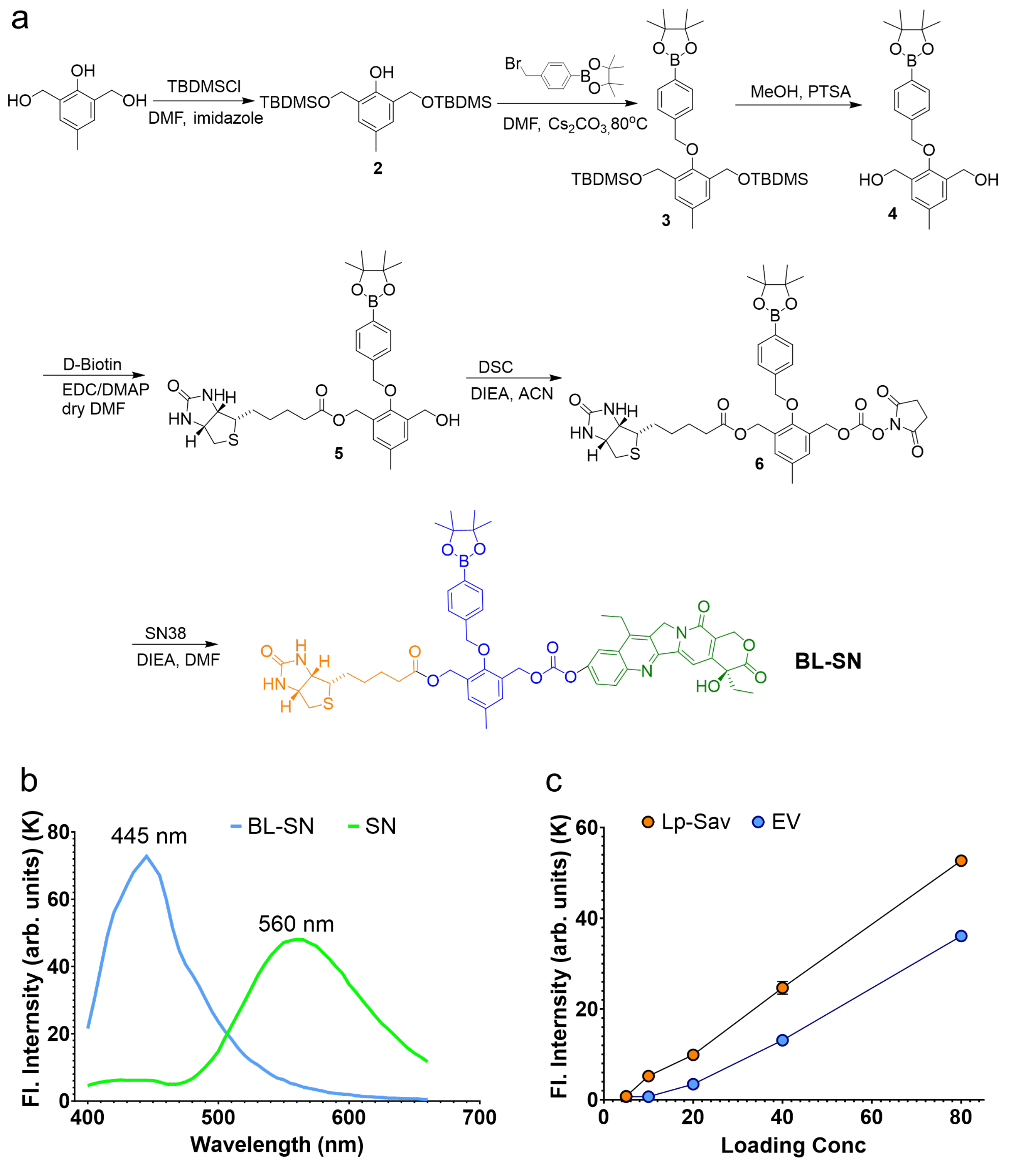

Supplementary Figure 15. Synthesis route of BL-SN and loading of BL-SN. a) Schematics showing the synthesis route of BL-SN and the loading of BL-SN. b) Fluorescence spectrum scanning showing the optimal emission wavelengths of BL-SN and SN. c) Loading of BL-SN on the Lp-Sav and EV strains. n=3 independent Lp cultures. Data are presented as mean values +/- SEM.

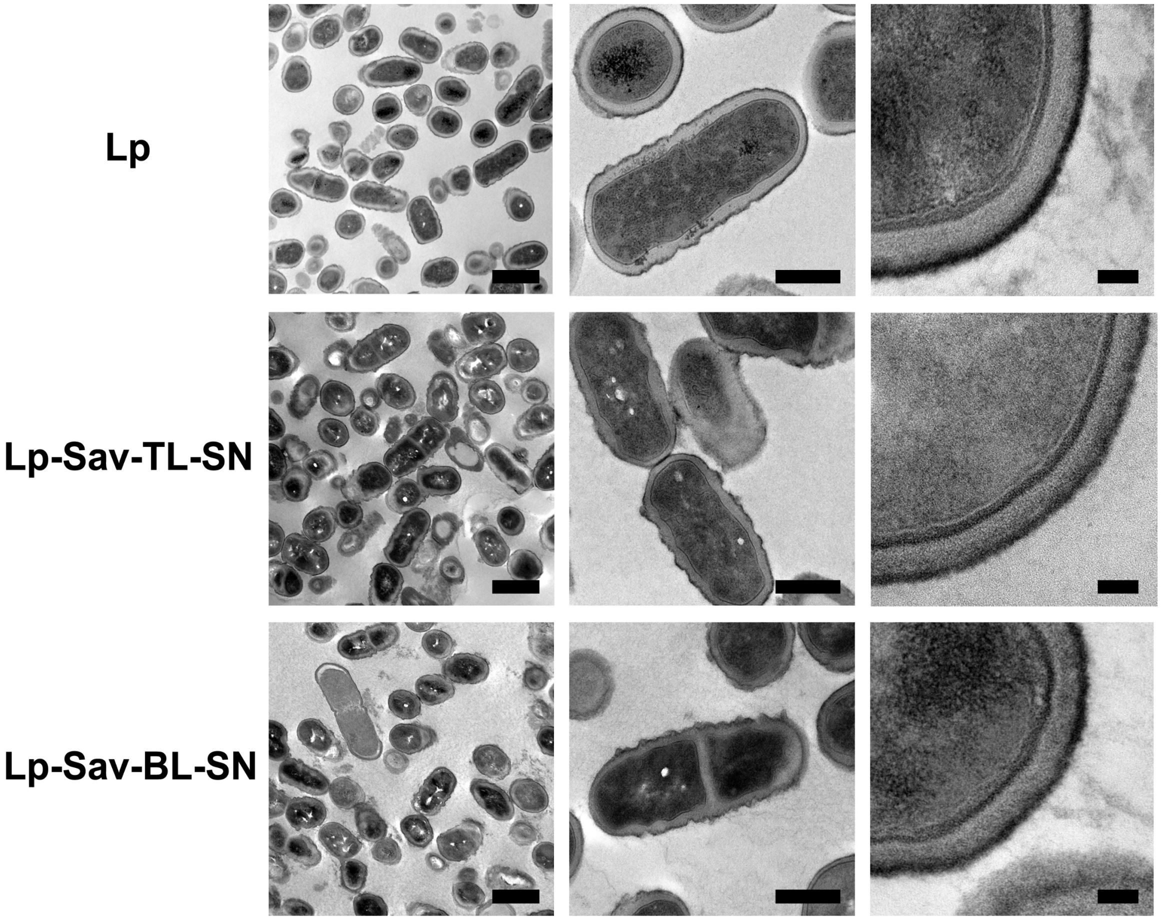

Supplementary Figure 16. TEM images showing the membrane structure of Lp, Lp-Sav-TL-SN and Lp-Sav-BL-SN. Scale bars from left to right, 1.0 μm, 200.0 nm, 50.0 nm.

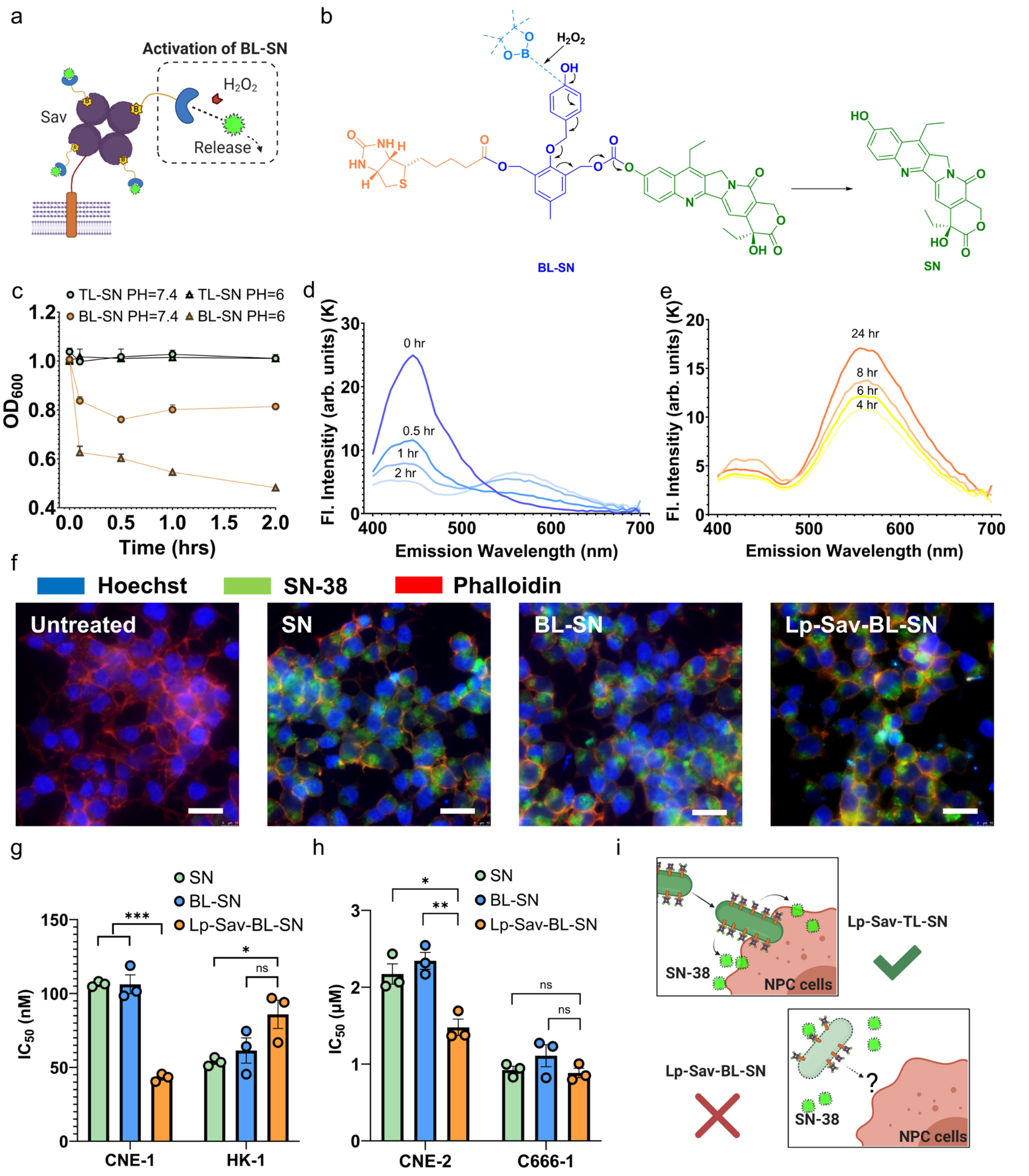

Supplementary Figure 17. Release of SN from Lp-Sav-BL-SN and in vitro characterization of Lp-Sav-BL-SN in NPC cells. a) & b) Schematics showing the mechanism of BL-SN activation and SN release. c) Bacterial cell lysis caused by activation of BL-SN. n=3 independent Lp cultures. d) & e) Release of SN from Lp-Sav-BL-SN via H_2O_2 activation detected by fluorescence. f) Accumulation of SN in C666-1 cells after Lp-Sav-BL-SN administration. 24 hours incubation time. Red – β-actin stained by phalloidin. Blue – nucleus stained by Hoechst. Green – SN. Scale bars, 25 μm. g) & h) IC_{50} of various treatments in NPC cells. n=3 independent NPC cultures. (P values in CNE-1 group SN vs Lp-Sav-BL-SN = 3.99×10^{-6}, BL-SN vs Lp-Sav-BL-SN = 0.0007; in HK-1 group 0.0298, 0.1287; in CNE-2 group 0.0151, 0.0048; in C666-1 group 0.6439, 0.2243). i) Schematics showing that the lysis of cells led to nontargeted release of SN in Lp-Sav-BL-SN cells. Data are presented as mean values +/- SEM.

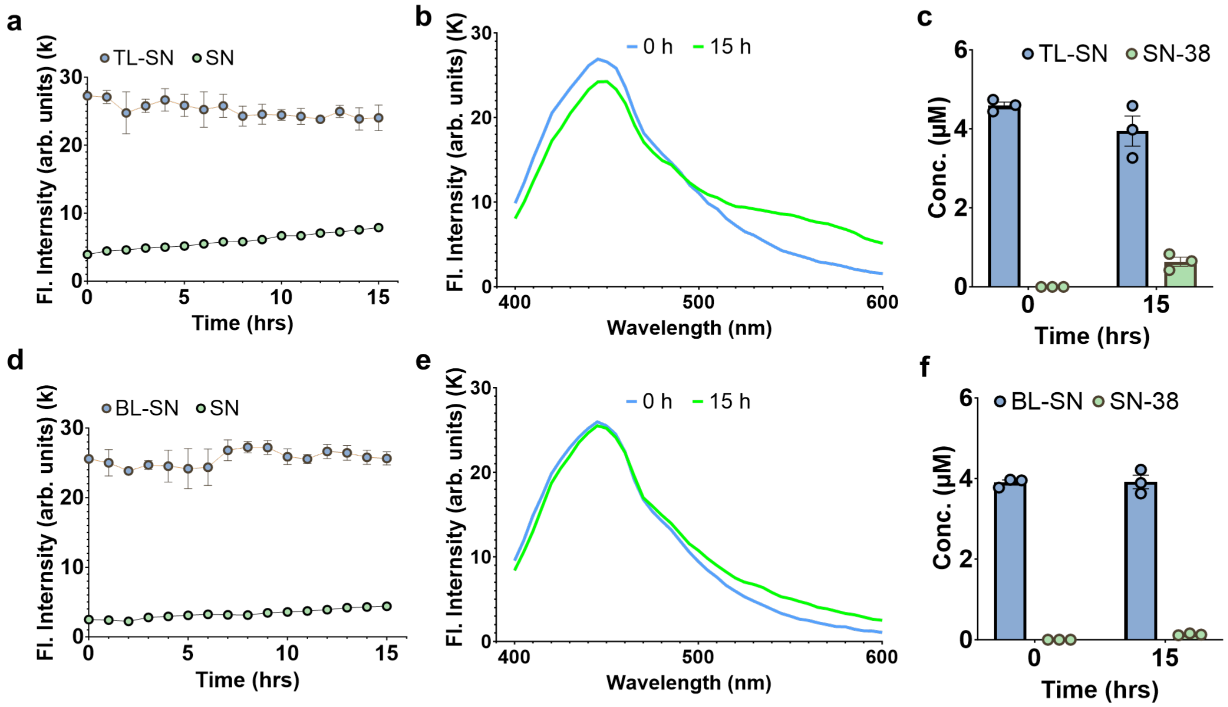

Supplementary Figure 18. Stability of Lp-Sav-TL-SN and Lp-Sav-BL-SN in PBS. a) & d) Activation of TL-SN and BL-SN with the release of SN from TL-SN-loaded and BL-SN-loaded Lp-Sav over time, respectively. b) & e) Dynamics of TL-SN and BL-SN activation, respectively by spectrum scanning over time. c) & f) Concentration of TL-SN and BL-SN in the cell pellet, respectively and SN in the supernatant pre- and post-activation. The assays are performed in 3 independent Lp cultures. Data are presented as mean values +/- SEM.

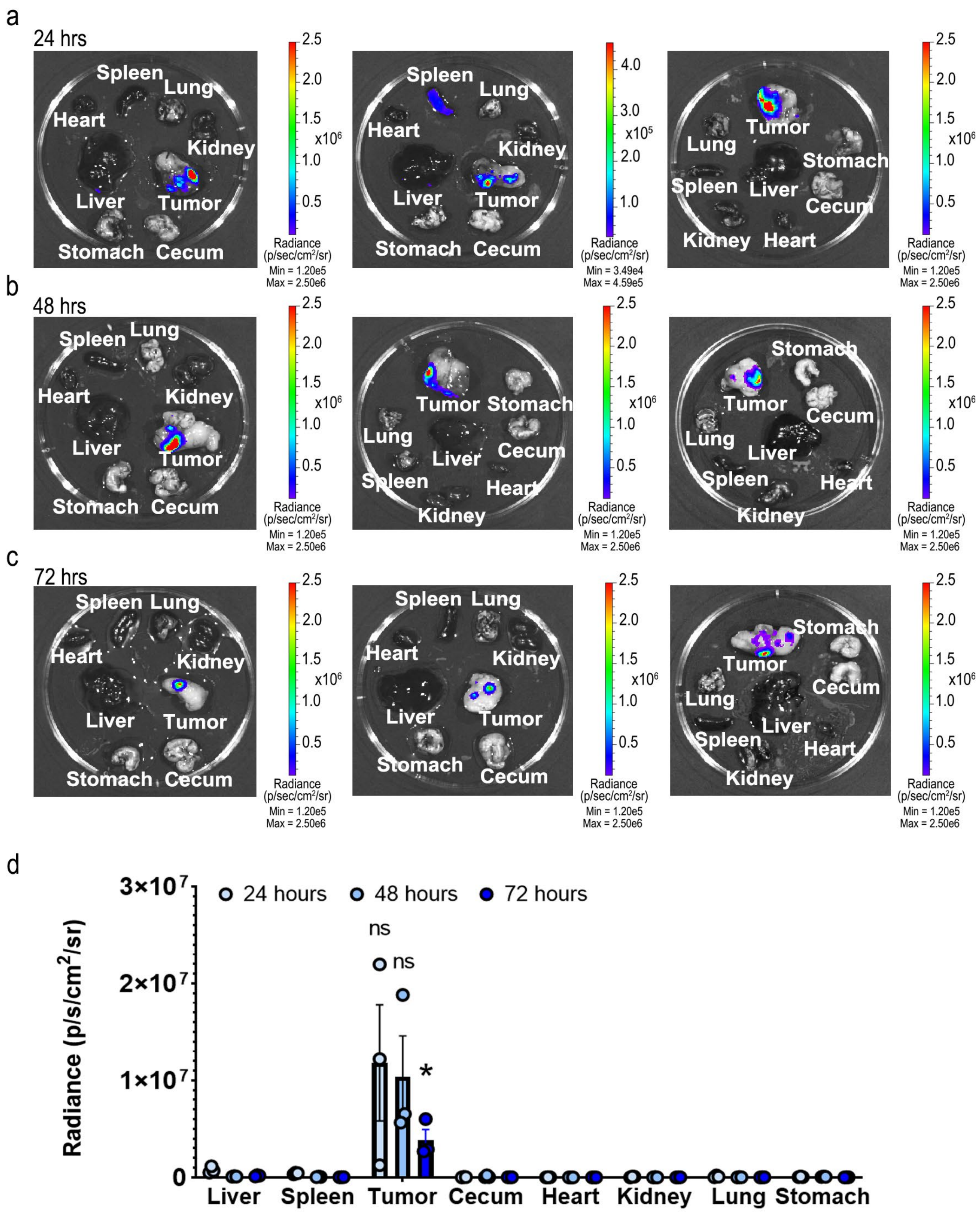

Supplementary Figure 19. IVIS analysis of bioluminescent Lp-CB in mouse organs 72 hours post intravenous injection. a), b) & c) IVIS imaging of the mouse organs 24, 48, 72 hours post injection. d) Quantification of bioluminescence from mouse organs 24, 48, 72 hours post injection. n=3 mice. Data are presented as mean values +/- SEM. P values vs Liver = 0.0271, vs Spleen =0.0240, vs Cecum = 0.0239, vs Heart = 0.0236, vs Kidney = 0.0237, vs Lung = 0.0239, vs Stomach = 0.0239.

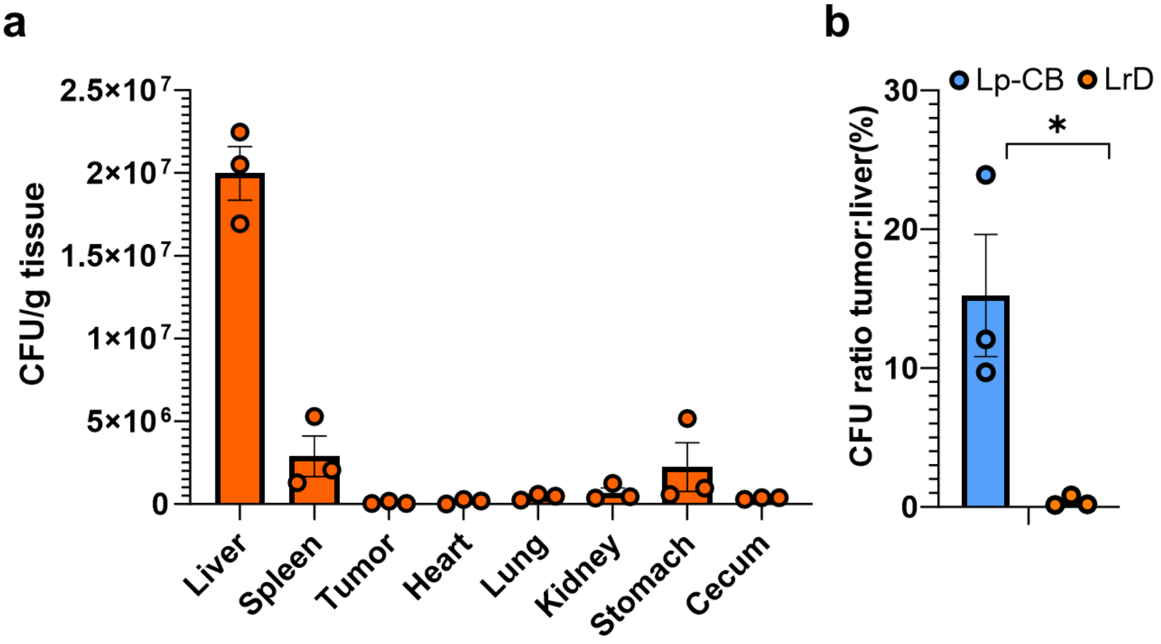

Supplementary Figure 20. Biodistribution of LrD in mouse following intravenous injection. a) CFU cout of LrD in various organs 24 hour following administration. n=3 mice. b) Tumor-to-liver ration of Lp-CB and LrD 24 hours postinjection. (p value = 0.0281). n=3 mice. Data are presented as mean values +/- SEM.

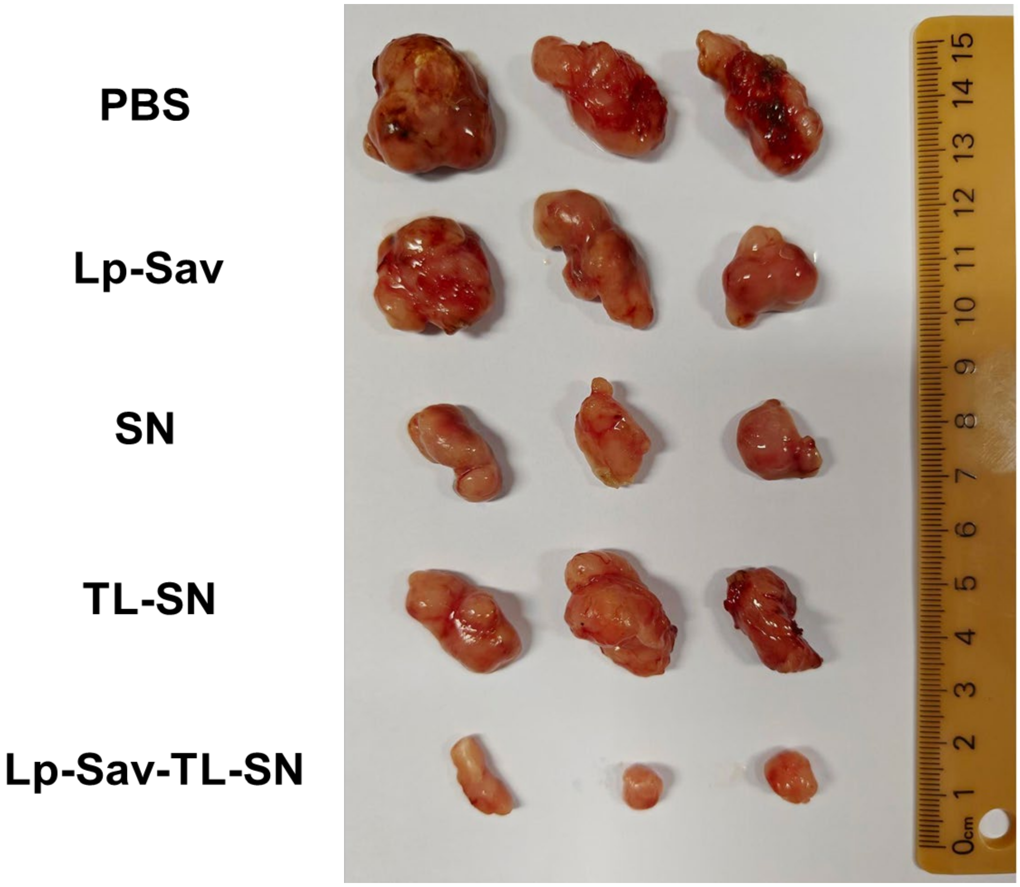

Supplementary Figure 21. Representative C666-1 tumors excised from various treatment groups.

Supplementary Table 1: Hydrogen bond interacting sites of different Lp_0018-heparin complexes

Pose	Binding site	Hydrogen bond interacting side chains
0	1	His98, Lys 80, Asn99, Lys 101, Arg 232, Lys 233, Gln 234, Lys 81, Arg 97, Asn 69, Lys 71, Arg 533, Asp 231
1	1	Arg97, Asn99, Lys 81, Asn 69, Arg 533, Gln 234, Arg 232, Lys 101, Asp 231, Lys 233, Arg 236.
2	1	Arg 232, Arg 97, His98, Lys 80, Asn 99, Lys 81, Lys 233 and lys 101
3	1	Lys 81, Lys 71, Gln 70, Asn 6, Arg 97, Asn 99, Lys 233, Gln 234, Arg 533, Asp 231
4	2	Lys 480, Lys 147, Gln 167, Lys 155, Gln 163, Asn88, Thr 86, Lys 84, Thr 93, Lys 90, Lys 137, Thr 91, Thr 165, Asn 138
5	1	Lys 107, Lys 101, Arg 232, Lys 233, Gln 234, Arg 97, Asn99, lys 81, Asp231
6	2	Arg 169, Gln 481, Arg 486, Tyr 172, lys 480, His 132, Lys90, Gln 168, Gln 167, Lys 137, lys 147
7	1	Lys 159, lys 161, Asn95, Lys 80, Arg 97, Tyr 160, Lys 101, Lys 107
8	2	Gln 481, Arg 169, Lys 480, Gln 168, Lys 137, Lys 90, Asn 88, Lys 84, Thr 93, Lys 155, Gln 163, Thr 91
9	1	Lys 107, Lys 80, Lys 233, Arg 232, Lys 101
10	1	Asn95, Lys 161, Lys 159, Lys 101, His 98, Arg 97, Lys 80
11	1	Lys 161, Asn 95, Lys80, His 98, Arg 97, Lys 81, Arg 533, Arg 232, Lys 233
12	1	Tyr 160, lys 159, Lys 107, Tyr 288, Lys 106, Lys 191 and Asn 104
13	2	Lys 480, Arg 169,Gln 481, Thr 477, Thr 140, Arg 176, Asn 451, Glu 470, Tyr 172, Ser 454, Thr 483.
14	1	Lys 159, Thr 109, Gln 187, Asn 185, Lys 107, His 98, Lys 101
15	1	Gln163, Lys 155, Lys 84, Lys 161, Lys 90, Lys 137
16	1	Asn 174, Tyr66, Tyr 175, Gln 444, Lys 71, Lys 81, Arg 533, Asn 69 and Arg 97
17	1	Lys 161, Lys 84, Asn95, Lys 80, His 98

Supplementary Table 2: Rearrangement of binding pose based on binding orientation with different OppA proteins

Binding complex	**Lp_0018**	**Lp_0092**	**Lp_0200**	**Lp_0201**	**Lp_0783**	**Lp_1261**	**Lp_3686**
Complex 0	Site 1	Site 2	Site 2	Site 2	Site 3	Site 1	Site 2
Complex 1	Site 1	Site 2	Site 2	Site 2	Site 3	Site 1	Site 2
Complex 2	Site 1	Site 2	Random	Site 2	Site 3	Site 1	Site 2
Complex 3	Site 1	Site 2	Random	Site 4	Site 3	Site 1	Site 2
Complex 4	Site 2	Site 2	Random	Site 4	Site 3	Site 1	Site 2
Complex 5	Site 1	Site 1	Site 1	Site 2	Site 3	Site 1	Site 2
Complex 6	Site 2	Site 1	Site 1	Site 2	Site 3	Site 1	Site 1
Complex 7	Site 1	Site 2	Random	Site 4	Site 1	Site 1	Site 2
Complex 8	Site 2	Site 3	Site 1	Site 2	Site 3	Site 1	Site 1
Complex 9	Site 1	Site 1	Site 1	Site 4	Site 3	Site 1	Site 2
Complex 10	Site 1	Site 3	Random		Site 3	Site 1	Site 2
Complex 11	Site 1	Site 2	Random		Site 3	Site 1	Site 1
Complex 12	Site 1	Site 1	Site 2		Site 3	Random	Site 2
Complex 13	Site 2	Site 1	Site 1			Random	Site 2
Complex 14	Site 2	Site 2	Site 2			Site 1	
Complex 15	Site 1	Site 3	Site 1			random	
Complex 16	Site 1	Site 3	Random			Site 1	
Complex 17	Site 1	Site 3	Random				
Complex 18		Site 1	Random				
Complex 19		Site 1	Site 1				
Complex 20		Site 1	Site 1				

Supplementary Table 3: Sequence and purification conditions of OppA proteins

Lane	OppA Proteins	Uniprot Entry	Sequence	Purification Condition	Elution Condition
**1**	**Lp_0018 SBD**	F9US48	AA 70 to 538	LB media, 100µM IPTG, 18ºC incubation for 24 hours	1 M NaCl
**2**	**Lp_0018**	F9US48	AA 27 to 538	LB media, 100µM IPTG, 25ºC incubation for 24 hours	1 M NaCl
**3**	**Lp_0092**	F9USS1	AA 29 to 519	TB auto-induction media, 30ºC incubation for 24 hours	0.5 M NaCl
**4**	**Lp_0200**	F9UT07	AA 84 to 553	LB media, 100µM IPTG, 25ºC incubation for 24 hours	0.35 M NaCl (Further purification through nickel column)
**5**	**Lp_0201**	F9UT08	AA 39 to 553	LB media, 100µM IPTG, 18ºC incubation for 24 hours	0.9 M NaCl
**6**	**Lp_0783**	F9UM05	AA 21 to 555	LB media, 100µM IPTG, 25ºC incubation for 24 hours	0.7 M NaCl
**7**	**Lp_1261**	F9UN51	AA 31 to 547	LB media, 100µM IPTG, 25ºC incubation for 24 hours	0.8 M NaCl
**8**	**Lp_3686**	F9ULM7	AA 36 to 545	LB media, 100µM IPTG, 25ºC incubation for 24 hours	0.8 M NaCl

Supplementary Table 4: Promoters characterized in *L. plantarum* WCFS1

**Promoters**	**Origin and Source references**
P32	*Lactococcus lactis* subsp. lactis N8 [1]
Ldhs	*Lactobacillus sakei* [2]
Ldhp	*Lactobacillus plantarum* WCFS1 [3]
SlpA	*Lactobacillus acidophilous* [4]
PGM	*Lactobacillus acidophilous* [5]
P11	Synthetic promoter for *L. plantarum* [6]
Slp	*Lactobacillus acidophilous* [7]
Puo19	*Lactobacillus casei phage* [8]
ermB	Promoter of Erythromycin resistance gene [7]
P8	*Lactococcus lactis* subsp. lactis N8 [9]

References

1 Cameron, D. E. & Collins, J. J. Tunable protein degradation in bacteria. *Nat Biotechnol* **32**, 1276-1281, (2014). https://doi.org/10.1038/nbt.3053

2 Bao, S. *et al.* Distribution dynamics of recombinant Lactobacillus in the gastrointestinal tract of neonatal rats. *PLoS One* **8**, e60007, (2013). https://doi.org/10.1371/journal.pone.0060007

3 Sasikumar, P. *et al.* Genetically engineered Lactobacillus plantarum WCFS1 constitutively secreting heterologous oxalate decarboxylase and degrading oxalate under in vitro. *Curr Microbiol* **69**, 708-715, (2014). https://doi.org/10.1007/s00284-014-0644-2

4 McCracken, A., Turner, M. S., Giffard, P., Hafner, L. M. & Timms, P. Analysis of promoter sequences from Lactobacillus and Lactococcus and their activity in several Lactobacillus species. *Arch Microbiol* **173**, 383-389, (2000). https://doi.org/10.1007/s002030000159

5 Duong, T., Miller, M. J., Barrangou, R., Azcarate-Peril, M. A. & Klaenhammer, T. R. Construction of vectors for inducible and constitutive gene expression in Lactobacillus. *Microb Biotechnol* **4**, 357-367, (2011). https://doi.org/10.1111/j.1751-7915.2010.00200.x

6 Rud, I., Jensen, P. R., Naterstad, K. & Axelsson, L. A synthetic promoter library for constitutive gene expression in Lactobacillus plantarum. *Microbiology (Reading)* **152**, 1011-1019, (2006). https://doi.org/10.1099/mic.0.28599-0

7 Lizier, M., Sarra, P. G., Cauda, R. & Lucchini, F. Comparison of expression vectors in Lactobacillus reuteri strains. *FEMS Microbiol Lett* **308**, 8-15, (2010). https://doi.org/10.1111/j.1574-6968.2010.01978.x

8 García, P., Bascarán, V., Rodríguez, A. & Suárez, J. E. Isolation and characterization of promoters from the Lactobacillus casei temperate bacteriophage A2. *Can J Microbiol* **43**, 1063-1068, (1997). https://doi.org/10.1139/m97-151

9 Zhu, D. *et al.* Isolation of strong constitutive promoters from Lactococcus lactis subsp. lactis N8. *FEMS Microbiol Lett* **362**, (2015). https://doi.org/10.1093/femsle/fnv107
