# Supplementary material for: Prodrug-conjugated tumor-seeking commensals for targeted cancer therapy
Source: Nat Commun. 2024 May 21;15:4343. doi: 10.1038/s41467-024-48661-y (PMC11109227; doi:10.1038/s41467-024-48661-y)
Supplement: Supplementary file 3 — Reporting Summary [file 41467_2024_48661_MOESM3_ESM.pdf]

nature portfolio

Corresponding author(s): Chang, Matthew Wook

Last updated by author(s): Mar 15, 2024

Reporting Summary

Nature Portfolio wishes to improve the reproducibility of the work that we publish. This form provides structure for consistency and transparency in reporting. For further information on Nature Portfolio policies, see our Editorial Policies and the Editorial Policy Checklist.

Statistics

For all statistical analyses, confirm that the following items are present in the figure legend, table legend, main text, or Methods section.

n/a	Confirmed	
☐	☒	The exact sample size (*n*) for each experimental group/condition, given as a discrete number and unit of measurement
☐	☒	A statement on whether measurements were taken from distinct samples or whether the same sample was measured repeatedly
☐	☒	The statistical test(s) used AND whether they are one- or two-sided<br>*Only common tests should be described solely by name; describe more complex techniques in the Methods section.*
☐	☒	A description of all covariates tested
☐	☒	A description of any assumptions or corrections, such as tests of normality and adjustment for multiple comparisons
☐	☒	A full description of the statistical parameters including central tendency (e.g. means) or other basic estimates (e.g. regression coefficient) AND variation (e.g. standard deviation) or associated estimates of uncertainty (e.g. confidence intervals)
☐	☒	For null hypothesis testing, the test statistic (e.g. *F*, *t*, *r*) with confidence intervals, effect sizes, degrees of freedom and *P* value noted<br>*Give P values as exact values whenever suitable.*
☒	☐	For Bayesian analysis, information on the choice of priors and Markov chain Monte Carlo settings
☒	☐	For hierarchical and complex designs, identification of the appropriate level for tests and full reporting of outcomes
☒	☐	Estimates of effect sizes (e.g. Cohen's *d*, Pearson's *r*), indicating how they were calculated

Our web collection on statistics for biologists contains articles on many of the points above.

Software and code

Policy information about availability of computer code

Data collection	Bioteck Gen5, IVIS spectrum (PerkinElmer), Leica Las X, CytExpert 2.4 (Beckman), Cluspro 2.0
Data analysis	Microsoft Excel (Office 365), Graphpad Prism 10, Flowjo Vx and PyMOL (2.5.4)

For manuscripts utilizing custom algorithms or software that are central to the research but not yet described in published literature, software must be made available to editors and reviewers. We strongly encourage code deposition in a community repository (e.g. GitHub). See the Nature Portfolio guidelines for submitting code & software for further information.

Data

Policy information about availability of data

All manuscripts must include a data availability statement. This statement should provide the following information, where applicable:

- Accession codes, unique identifiers, or web links for publicly available datasets
- A description of any restrictions on data availability
- For clinical datasets or third party data, please ensure that the statement adheres to our policy

All data supporting the findings of this study are available in the article and the supplementary information file. Source data are provided as a Source Data file.

Research involving human participants, their data, or biological material

Policy information about studies with human participants or human data. See also policy information about sex, gender (identity/presentation), and sexual orientation and race, ethnicity and racism.

Reporting on sex and gender	NA
Reporting on race, ethnicity, or other socially relevant groupings	NA
Population characteristics	NA
Recruitment	NA
Ethics oversight	NA

Note that full information on the approval of the study protocol must also be provided in the manuscript.

Field-specific reporting

Please select the one below that is the best fit for your research. If you are not sure, read the appropriate sections before making your selection.

☒ Life sciences ☐ Behavioural & social sciences ☐ Ecological, evolutionary & environmental sciences

For a reference copy of the document with all sections, see nature.com/documents/nr-reporting-summary-flat.pdf

Life sciences study design

All studies must disclose on these points even when the disclosure is negative.

Sample size	ample sizes for the animal study were selected based on previous studies (https://doi.org/10.1016/j.colsurfb.2020.111223; https://pubs.acs.org/doi/10.1021/acs.bioconjchem.2c00559.)
Data exclusions	No data were excluded from the analyses
Replication	In vitro and in vivo experiments were performed in at least 3 biological replicates. All animal experiments were performed with 3 or 5 mice per group.
Randomization	Biological specimens and mice were randomly allocated into treatment groups.
Blinding	Chemical synthesis and analysis, formulation of the treatment groups and treatment of the mice were conducted by different individuals.

Reporting for specific materials, systems and methods

We require information from authors about some types of materials, experimental systems and methods used in many studies. Here, indicate whether each material, system or method listed is relevant to your study. If you are not sure if a list item applies to your research, read the appropriate section before selecting a response.

Materials & experimental systems

n/a	Involved in the study	
☐	☒	Antibodies
☐	☒	Eukaryotic cell lines
☒	☐	Palaeontology and archaeology
☐	☒	Animals and other organisms
☒	☐	Clinical data
☒	☐	Dual use research of concern
☒	☐	Plants

Methods

n/a	Involved in the study	
☒	☐	ChIP-seq
☐	☒	Flow cytometry
☒	☐	MRI-based neuroimaging

Antibodies

Antibodies used	Primary antibodies: Alexa Fluor 488 phalloidin (Abcam, ab176753); anti-GAPDH mouse monoclonal antibody (Cell Signaling, CST #97166); acetylated α-tubulin rabbit monoclonal antibody (Abcam, ab209348). mouse monoclonal anti-cMyc antibody (Thermo Fisher MA1-980); mouse monoclonal anti-HA antibody (Thermo Fisher 26183), rabbit monoclonal anti-syndecan-1 antibody (Abcam

	ab128936); rabbit polyclonal anti-syndecan-2 antibody (Abcam ab205884). Secondary antibodies Anti-rabbit IgG (CST 8889S Alexa Fluor 594 Conjugate, CST 4412S Alexa Fluor 488 Conjugate), Anti-mouse IgG (CST 8890S Alexa Fluor 594 Conjugate, CST 4408S Alexa Fluor 488 Conjugate), anti-mouse IgG HRP conjugate CST #7076.
Validation	Manufacturer validation data for flow cytometric/immunofluorescence/western blot analysis in Pubmed: 29301106, 15769908, 30260431, 12604612, 31273573, 31914402

Eukaryotic cell lines

Policy information about cell lines and Sex and Gender in Research

Cell line source(s)	RPMI 2650 cells were purchased from CLS (Cell Line Services) Germany. A549, AGS, and T24 cells were purchased from ATCC (American Type Culture Collection), America. CNE-1 and CNE-2 cells were kindly provided by A/Prof Shen Han Ming from the Department of Physiology, NUS. C666-1 cells were kindly provided by Dr. Joshua Tay from the Department of Otolaryngology, NUS. Human healthy nasal cells (HNCs) were a kind gift from Prof. Wang De Yun from the Department of Otolaryngology, NUS.
Authentication	RPMI 2650 cells were authenticated by CLS; A549, AGS, and T24 cells were authenticated by ATCC; CNE-1, CNE-2, HK-1, C666-1 and HNC cells were verified in previous publications, Pubmed: 33863904, 35394843, 22856354
Mycoplasma contamination	All cell line tested negative for mycoplasma
Commonly misidentified lines (See ICLAC register)	Among the cell lines, CNE-1 and CNE-2 were identified as misidentified cell lines maintained by the International Cell Line Authentication Committee. Previous reports provided detailed authentication of both cell lines, which identified distinctive NPC cell genomes alongside genomic contamination from HeLa cells through short tandem repeat profiling77. In this study, we identified CNE-1 and CNE-2 cells expressing HSPG syndecan-1 and syndecan-2, rendering them suitable targets for studying the binding between Lp and NPC cells.

Animals and other research organisms

Policy information about studies involving animals; ARRIVE guidelines recommended for reporting animal research, and Sex and Gender in Research

Laboratory animals	Balb/c nude mice, aged 4-5 weeks, weight 18–22 g, JAX® Mice, male, maintained under standard housing conditions (12 light/12 dark cycle, 22–24 °C with humidity set at 40–50%).
Wild animals	NA
Reporting on sex	Male
Field-collected samples	NA
Ethics oversight	National University of Singapore Institutional Animal Care and Use Committee (Protocol R21-1009)

Note that full information on the approval of the study protocol must also be provided in the manuscript.

Plants

Seed stocks	NA
Novel plant genotypes	NA
Authentication	NA

Flow Cytometry

Plots

Confirm that:

☒ The axis labels state the marker and fluorochrome used (e.g. CD4-FITC).

☒ The axis scales are clearly visible. Include numbers along axes only for bottom left plot of group (a 'group' is an analysis of identical markers).

☐ All plots are contour plots with outliers or pseudocolor plots.

☐ A numerical value for number of cells or percentage (with statistics) is provided.

Methodology

Sample preparation	NPC cells were scraped using cell scrapers and fixed with 4% PFA. The fixed cells were incubated with the OppA proteins overnight at 4°C and then incubated with a c-Myc monoclonal antibody (Thermo Fisher MA1-980) overnight at 4°C. The cells were then incubated with a secondary anti-mouse IgG (Alexa Fluor 488, Cell Signaling 4408) for one hour and analyzed in the CytoFLEX analyzer (Beckman).
Instrument	CytoFLEX analyzer (Beckman)
Software	FlowJo Vx
Cell population abundance	Whole cell population were analyzed and the geometric means of the treated cells were used to compare the binding efficacy of OppA proteins to various NPC lines.
Gating strategy	NA

☒ Tick this box to confirm that a figure exemplifying the gating strategy is provided in the Supplementary Information.
